# Quantifying the risk of rabies in biting dogs in Haiti

**DOI:** 10.1038/s41598-020-57908-9

**Published:** 2020-01-23

**Authors:** Xiaoyue Ma, Jesse D. Blanton, Max Francois Millien, Alexandra M. Medley, Melissa D. Etheart, Natael Fénelon, Ryan M. Wallace

**Affiliations:** 1 Centers for Disease Control and Prevention, Atlanta, GA 30329 USA; 2 Ministry of Agriculture and Natural Resources and Rural Development, Port-au-Prince, Haiti; 3 Centers for Disease Control and Prevention in Haiti, Division of Global Health and Prevention, Tabarre 41, Boulevard 15 Octobre, Port-au-Prince, Haiti; 4 Pan American Health Organization, Port-au-Prince, Haiti

**Keywords:** Infectious diseases, Risk factors

## Abstract

Rabies is a fatal viral disease typically transmitted through the bite of rabid animal. Domestic dogs cause over 99% of human rabies deaths. Over half of the world’s population lives in a country where the canine rabies virus variant is endemic and dog bites are common. An estimated 29 million people worldwide receive post-bite vaccination after being exposed to animals suspected of rabies. Accurate and timely risk assessment of rabies in biting dogs is critical to ensure that rabies PEP is administered to all persons with a suspected rabies exposure, while avoiding PEP administration in situations where rabies can be definitively ruled out. In this study, a logistic regression model was developed to quantify the risk of rabies in biting dogs, using data from Haiti’s animal rabies surveillance program. Significant risk factors identified in the model were used to quantify the probability of rabies in biting dogs. The risk of rabies in a biting dog as assessed through Haiti’s rabies surveillance program was highly elevated when the dog displayed hypersalivation (OR = 34.6, 95% CI 11.3–106.5) or paralysis (OR = 19.0, 95% CI 4.8–74.8) and when the dog was dead at the time of the assessment (OR = 20.7, 95% CI 6.7–63.7). Lack of prior rabies vaccination, biting 2 or more people, and if the dog was a puppy also increased the probability that a biting dog would have rabies. The model showed high sensitivity (100%) and specificity (97%) when examined using validation data. This model enables us to project the risk of rabies in biting dogs in Haiti shortly after the bite event and make provisional PEP recommendations prior to laboratory testing or dog quarantine results. Application of this model may improve adherence to PEP for bite victims who can be educated on the quantitative risk of the exposure event. This model can also be used to reduce unnecessary PEP costs when the risk of rabies is determined as sufficiently low and the animal is available for observation.

## Introduction

Rabies is an invariably fatal viral zoonosis typically transmitted through the bite of an infected animal^1^. Globally, domestic dogs are responsible for over 99% of the 59,000 human rabies deaths estimated to occur each year^2,3^. Over half of the world’s population lives in a country where the canine rabies virus variant (CRVV) is endemic. Rabies is preventable with timely washing of the wound and administration of post-exposure prophylaxis (PEP), which consists of rabies immune globulin (RIG) and a series of four doses of vaccine over a 14-day period. It is estimated that over 29 million rabies vaccine doses are administered in canine rabies endemic countries each year, at a cost of 1.7 billion USD^2^. The cost to treat a rabies exposure in most developing countries is in excess of one month’s salary, a substantial financial burden for many bite victims^4–6^. Furthermore, treatment typically requires four to five doctor visits, which is an additional financial and logistical burden.

More than 29 million people worldwide receive post-bite rabies vaccination each year for dog bites, yet rabies continues to cause more deaths than any other zoonotic disease^1,2,6^. Rabies deaths are often attributed to lack of access to timely vaccination and lack of knowledge about the necessity of prompt medical care. Access can be limited due to financial or logistical restraints. Interventions focused on increasing bite victim’s awareness of the risk of rabies have shown to improve adherence to PEP recommendations. Integrated Bite Case Management (IBCM) programs, programs that combine field assessment of rabies suspect animals to inform victims and PEP recommendations, have been implemented in several CRVV endemic settings such as Philippines, India, and Haiti^7,8^. IBCM programs typically utilize veterinary professionals to assess the health of/ or likely rabies status of biting dogs and articulate the rabies risk to the victim, healthcare provider, and public health authorities. An IBCM program implemented in Haiti was credited with decreasing human rabies cases by 50% through improved bite-victim adherence to vaccination schedules, which was attributed to risk-based counseling^9^.

IBCM programs have traditionally focused on identifying rabid dogs and improving PEP adherence among persons with likely rabies exposures. In 2018, the World Health Organization (WHO) published guidance describing the relationship between delayed PEP treatment and IBCM capacity; recommending that under certain low-risk exposure scenarios when highly qualified staff are available to assess the offending animal, PEP administration can be delayed while the animal is placed under evaluation^10^. Dog bites are exceedingly common, with many studies reporting dog bite rates in the range of one to three for every 100 persons per year. The majority of these bites are a result of human-derived antagonism and fear-based behaviors^11–13^. Several studies have reported rabies rates in biting dogs of less than 10%^5,9^. In many parts of the world diagnostic capacity and public health risk assessments are not available and rabies must be considered a possibility for every bite event. This may lead to unnecessary, burdensome expenses for bite victims and supply shortages.

Two publications have reported on the characteristics and estimated probabilities of rabies in biting dogs^14,15^. A Thai study developed a highly sensitive and specific model that took into account the characteristics of dog bite events and outcomes of 10-day quarantine periods^16^. Due to the inclusion of the quarantine outcome, this model cannot be used to assess the necessity for PEP at the time of the bite, and therefore is unlikely to substantially reduce the initiation of unnecessary PEP among bite victims. In 2017, a univariate analysis describing probability of rabies in biting dogs in Haiti found that several variables were highly associated with rabid dogs. However, no single variable displayed an enough sensitivity to recommend its utility in PEP recommendations. Here, we developed multivariable logistic regression models to quantify rabies risk in biting dogs using only information available at the time of an IBCM investigation and PEP risk assessment, but not including the outcome of a 10-day quarantine period. The sensitivity and specificity of the model predictions were assessed.

## Materials and Methods

### Data description

Data analyzed for this study were made available from the Haiti Ministry of Agriculture from the national animal rabies surveillance program (HARSP) database (2014–2016). The data source used in this study was previously described by Medley *et al*.^14^. At the conclusion of a rabies investigation, biting dogs assessed by the national surveillance program are classified into one of four categories: 1-confirmed case, 2-probable case (clinical case definition), 3-suspect case (unavailable for assessment), 4-case negative (14-day quarantine or negative direct fluorescent antibody result). For surveillance case definitions refer to Box 1 in Wallace *et al*.^7^. The final cohort analyzed for model development comprised 48 rabies positive dogs and 1,361 dogs for which rabies was ruled out (total n = 1,409). An independent data set consisting of confirmed (n = 5) and non-cases (n = 463) from 2016 were used for model evaluation.

### Model specification and evaluation

For the fitting datasets, the rabies status of the dogs is a discrete event, either the confirmed rabies case or non-rabies case. In this study, the dichotomous dependent variable was coded as 0 for non-rabies cases and 1 for the confirmed rabies cases. The predicted probability of rabies case should be in the range [0, 1]. The logistic function appears to be a good equation for predicting the probability of a dog that could be rabies case. The logistic model was formulated as

1 $$p={(1+{e}^{-X\beta })}^{-1}.$$

And the general form of log odds of rabies is given by

2 $$\log \left(\frac{p}{1-p}\right)={\bf{X}}{\boldsymbol{\beta }}$$

where *p* represents the probability that the dog is rabid (i.e. the probability that the dog does not have rabies is given by 1−*p*), ***X*** a vector of explanatory variables which characterize the dog information, **β** the vector of parameters to be estimated, and *e* is the base of the natural logarithm.

The potential explanatory variables (***X)*** considered in this analysis were collected on a standardized risk assessment tool conducted by Haiti’s Ministry of Agriculture Rural Development and Natural Resources^7^. These variables included the reporting source, the biting dog’s ownership status, age, sex, presence of hypersalivation, presence of paralysis, presence of lethargy, presence of aggression, vaccination status, and the current health status of the dog (alive vs dead), which were routinely collected during IBCM investigations. Furthermore, the subjective opinion of the IBCM investigator recorded for each case was also considered as a potential explanatory variable. A series of dummy variables were set to distinguish the different levels for each potential variable:

$$RE{P}_{1}=\left\{\begin{array}{ll}1& {\rm{Veterinary}}\,{\rm{Sectors}}\\ 0& {\rm{Otherwise}}\end{array}\right.$$

$$RE{P}_{2}=\left\{\begin{array}{ll}1&{\rm{Public}}\\ 0& {\rm{Otherwise}}\end{array}\right.$$

$$OWNED=\left\{\begin{array}{ll}1&{\rm{Stray}}\,{\rm{or}}\,{\rm{Unknown}}\\ 0&{\rm{Owned}}\,\end{array}\right.$$

$$BITES=\left\{\begin{array}{ll}1&{\rm{Bite}}\ge 2\\ 0&{\rm{Bite}}=1\end{array}\right.$$

$${SEX}_{1}=\left\{\begin{array}{cc}1&{\rm{Male}}\\0 & {\rm{Otherwise}}\end{array}\right.$$

$$SE{X}_{2}=\left\{\begin{array}{ll}1&{\rm{Unknown}}\,\\ 0&{\rm{Otherwise}}\,\end{array}\right.$$

$$AG{E}_{1}=\left\{\begin{array}{ll}1&{\rm{Puppy}}\,\\ 0&{\rm{Otherwise}}\end{array}\right.$$

$$AG{E}_{2}=\left\{\begin{array}{ll}1&{\rm{Junior}}\,\\ 0& {\rm{Otherwise}}\end{array}\right.$$

$$AG{E}_{3}=\left\{\begin{array}{ll}1& {\rm{Unknown}}\,\\ 0& {\rm{Otherwise}}\,\end{array}\right.$$

$$AGGRESSION=\left\{\begin{array}{ll}1&{\rm{Aggressive}}\\ 0& {\rm{Nonaggressive}}\end{array}\right.$$

$$HYPERSALIVATION=\left\{\begin{array}{ll}1& {\rm{Hypersalivation}}\\ 0& {\rm{Normal}}\,{\rm{salivation}}\end{array}\right.$$

$$PARALYZED=\left\{\begin{array}{ll}1& {\rm{Paralytic}}\\ 0& {\rm{Nonparalytic}}\end{array}\right.$$

$$LETHARGY=\left\{\begin{array}{ll}1& {\rm{Lethargic}}\\ 0& {\rm{Nonlethargic}}\end{array}\right.$$

$$NONVAX=\left\{\begin{array}{ll}1&{\rm{Not}}\,{\rm{vaccinated}}\\ 0&{\rm{Vaccinated}}\end{array}\right.$$

$$DEAD=\left\{\begin{array}{ll}1&{\rm{F}}{\rm{o}}{\rm{u}}{\rm{n}}{\rm{d}}\,{\rm{D}}{\rm{e}}{\rm{a}}{\rm{d}}\\ 0& {\rm{F}}{\rm{o}}{\rm{u}}{\rm{n}}{\rm{d}}\,{\rm{A}}{\rm{l}}{\rm{i}}{\rm{v}}{\rm{e}}\end{array}\right.$$

$$ASS{E}_{1}=\left\{\begin{array}{ll}1& {\rm{Probably}}\,{\rm{Rabies}}\\ 0&{\rm{Otherwise}}\end{array}\right.$$

$$ASS{E}_{2}=\left\{\begin{array}{ll}1& {\rm{Dead}}/{\rm{Not}}\,{\rm{Assessed}}\\ 0& {\rm{Otherwise}}\end{array}\right.$$

Maximum likelihood techniques were used to estimate the parameters of the model^17^. The most parsimonious model, in which all parameter estimates are significant, was selected based on the likelihood ratio test and the Hosmer-Lemeshow goodness-of-fit test (H-L test)^18^. A small *p*-value of the H-L test suggests that the fitted model is not an adequate model. If a function fits the data well, the p-value associated with that function should be larger than 0.05, indicating no significant deviation away from fitted function by the data at the 95% confidence level.

Two models were developed. Model I was built from the perspective of a beginning rabies program, and only considered standard variables from the assessment form, but did not consider the investigator’s assessment decision (*ASSE*_1_ and *ASSE*_2_). A second model (Model II) followed the aforementioned methodology, but also included the investigator’s assessment. This model was developed to assess the improvement in predicting the likelihood of rabies in a biting dog when considering the assessment decision of trained professionals. The investigator’s assessment opinion is based on some clinical information such as aggression, lethargy, paralysis, hypersalivation, etc. Although this does not imply that the opinion is (mathematically) highly correlated with these variables, we still explored whether the model is suffering the effects of multilinearity. Before fitting the models, multilinearity in the dataset was examined through the Variance Inflation Factor and Tolerance, and an eigensystem analysis of covariance comparison. All results indicate a lack of multilinearity in the dataset.

The confirmed rabies cases and non-cases from rabies assessments conducted in 2016 were used for validating Models I and II. The sensitivity and specificity for predicting rabies in dogs with Models I and II were evaluated using different probability levels as cutoff values.

### Model application

The developed logistic regression models (Models I and II) were used to predict the rabies status in dogs reported to Haiti’s animal rabies surveillance program during 2014–2016. The probability that a dog reported to Haiti’s program had rabies was estimated with Model I and Model II, and compared with a range of cutoff values. The dog was classified as a rabies case if its predicted probability was above the cutoff value; otherwise, it was a non-rabies case. Sensitivity and specificity were then calculated at different cut-off values for Models I and II.

**Figure 1:**
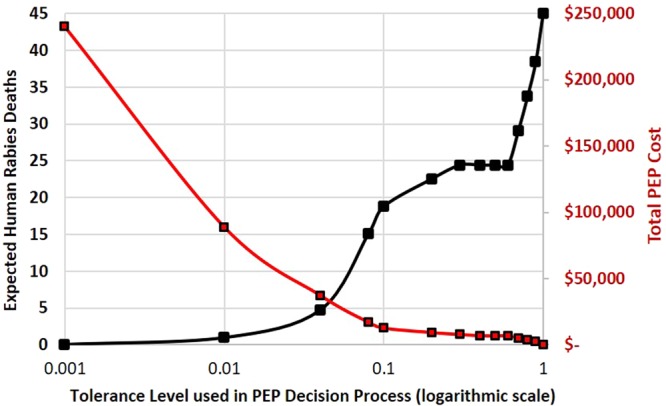
Expected human rabies deaths (black line) and total PEP costs (red line) by tolerance level for determining rabies status in biting dogs (Model I).

**Figure 2:**
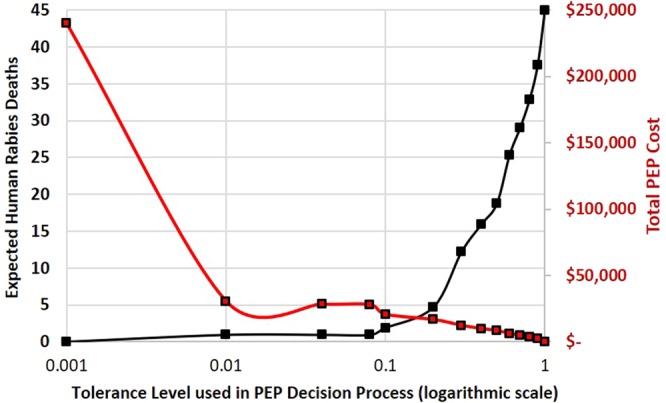
Expected human rabies deaths (black line) and total PEP costs (red line) by tolerance level for determining rabies status in biting dogs (Model II).

**Figure 3:**
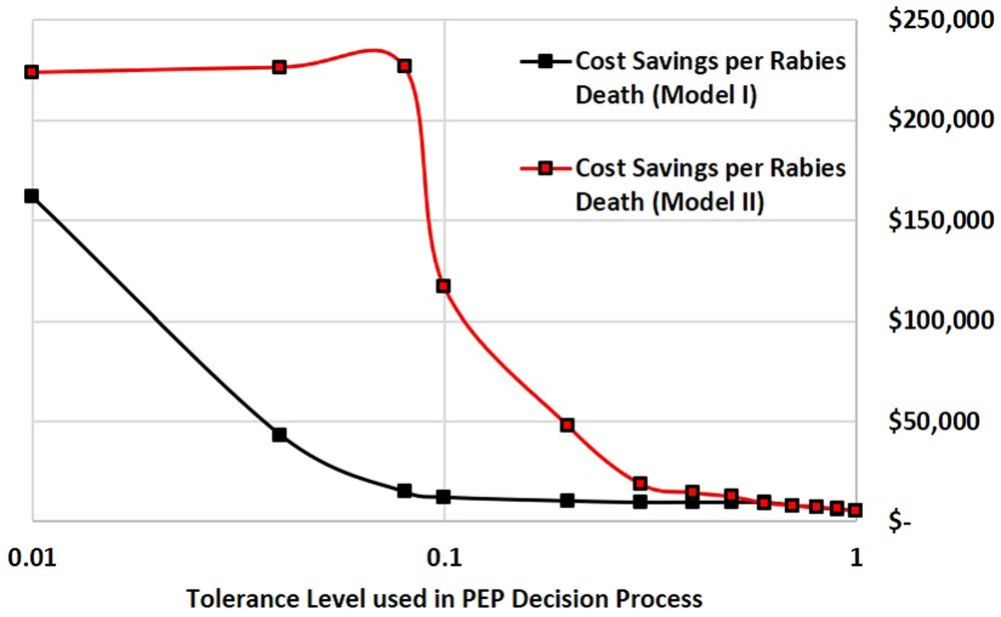
PEP cost-savings per human rabies death averted across tolerance levels for determining rabies status in biting dogs.

**Figure 4:**
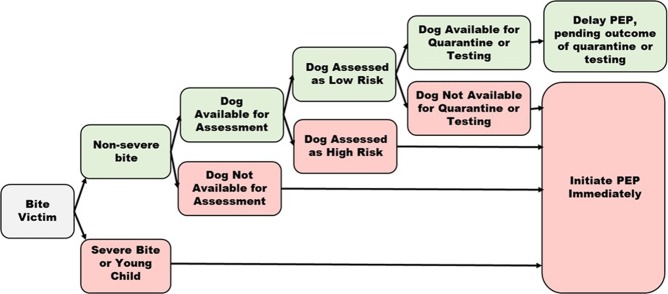
Algorithm for determining PEP recommendations based on risk assessment and quarantine outcomes, Haiti. (Severe bite includes bites to the head or multiple deep puncture wounds).

The prediction models were also used to estimate the likelihood that probable and suspect rabies cases truly were rabid, during the periods of 2014–2015 and 2016, respectively. A scenario was assessed based upon Haiti’s national rabies program, in which 5,000 bite victims were treated by the IBCM program (approximate average annual number of case investigations in Haiti from 2016–2018), and of which 5% were true rabies exposures (approximate average case positivity rate among investigated dogs in Haiti). The Model  was used to calculate the expected number of incorrectly predicted rabies cases (1 – sensitivity) and expected number of bite victims for whom PEP could be immediately delayed pending a 10-day quarantine or timely laboratory testing (specificity). The probability of human death given a true rabies exposure, in the absence of PEP, was 18% as obtained from Babes *et al*.^19^. A 4-dose intramuscular regimen, at a cost of $12 per vaccine dose (average cost in Haiti), was used to calculate the expected PEP cost, PEP cost-savings, and PEP cost per death averted under different probability scenarios (Figs. 1, 2 and 3). Finally, a proposed algorithm for PEP recommendations, which includes risk assessment and quarantine in the decision-making process, was developed (Fig. 4).

## Results

### Logistic regression model without the assessor’s assessment (Model I)

The explanatory variables that remained in the final model are listed in Table 1. The p-value of the H-L test for this model was 0.3978, indicating an adequate fit. The dog’s gender and ownership status were not significant predictors of the presence of rabies, nor was the presence of the clinical signs: aggression or lethargy. Adjusted odds ratio estimates for the final model are shown in Table 2. The probability that the biting dog had rabies was highly increased when the dog displayed hypersalivation (OR = 34.6, 95% CI 11.3–106.5) or paralysis (OR = 19.0, 95% CI 4.8–74.8) and when the dog was dead at the time of the assessment (OR = 20.7, 95% CI 6.7–63.7). Lack of prior rabies vaccination, biting two or more people, and if the dog was a puppy also increased the probability that the biting dog was rabid.

**Table 1:** Parameter estimates of the final model (Model I, without assessor’s assessment) for the probability of a dog that could be rabies case.

Variable	Parameter	Estimate	Standard error	*p*-Value
	${\hat{\beta }}_{0}$	−6.3845	0.7858	<0.0001
*BITES*	${\hat{\beta }}_{1}$	1.6788	0.4719	0.0004
*AGE*_1_	${\hat{\beta }}_{2}$	2.3651	0.6495	0.0003
*AGE*_2_	${\hat{\beta }}_{3}$	0.5269	0.4550	0.2469
*AGE*_3_	${\hat{\beta }}_{4}$	2.2633	0.5778	<0.0001
*HYPERSALIVATION*	${\hat{\beta }}_{5}$	3.5444	0.5733	<0.0001
*PARALYZED*	${\hat{\beta }}_{6}$	2.9458	0.6988	<0.0001
*NONVAX*	${\hat{\beta }}_{7}$	1.6293	0.7622	0.0325
DEAD*	${\hat{\beta }}_{8}$	3.0304	0.5731	<0.0001

*Indicates that the biting dog was dead at the time of IBCM investigation. Death may have occurred due to natural causes or killed by the community. The cause of death was not differentiated.

**Table 2:** Odds ratio estimates and 95% Confidence Limits (LCL, UCL) with Model I.

Variable	Odd Ratio	LCL	UCL
*BITES*	5.359	2.125	13.512
*AGE*_1_	10.645	2.981	38.017
*AGE*_2_	1.694	0.694	4.132
*AGE*_3_	9.615	3.098	29.837
*HYPERSALIVATION*	34.619	11.253	106.501
*PARALYZED*	19.025	4.836	74.845
*NONVAX*	5.100	1.145	22.719
*DEAD*	20.705	6.733	63.667

Model I:

$$log\left(\frac{p}{1-p}\right)={\hat{\beta }}_{0}+{\hat{\beta }}_{1}BITES+{\hat{\beta }}_{2}AG{E}_{1}+{\hat{\beta }}_{3}AG{E}_{2}+{\hat{\beta }}_{4}AG{E}_{3}+{\hat{\beta }}_{5}HYPERSALIVATION+{\hat{\beta }}_{6}PARALYZED+{\hat{\beta }}_{7}NONVAX+{\hat{\beta }}_{8}DEAD$$

### Logistic regression model with the assessor’s assessment (Model II)

The best set of explanatory variables including the assessor’s assessment and their parameter estimates are listed in Table 3. The p-value of the H-L test for this model was 0.8566. The dog’s sex, ownership status, and vaccination status were not significant predictors of the presence of rabies, nor was the presence of the clinical signs: aggression or lethargy. Odds ratio estimates for Model II are shown in Table 4. The most significant explanatory variable for predicting whether the biting dog had rabies was the subjective opinion of the investigator (OR 338.7). The clinical signs hypersalivation and paralysis also increased the probability the dog had rabies (OR 6.0 and 8.1, respectively). Dogs dead at the time of investigation, dogs that bit two or more people, and younger dogs also had higher probabilities of being rabid.

**Table 3:** Parameter estimates of the final model (Model II, with assessor’s assessment) for the probability of a dog that could be rabies case.

Variable	Parameter	Estimate	Standard error	*p*-Value
	${\hat{\beta }}_{0}$	−8.3047	1.0772	<0.0001
*BITES*	${\hat{\beta }}_{1}$	1.2372	0.5593	0.0270
*AGE*_1_	${\hat{\beta }}_{2}$	1.9918	0.7169	0.0055
*AGE*_2_	${\hat{\beta }}_{3}$	1.2859	0.5242	0.0142
*AGE*_3_	${\hat{\beta }}_{4}$	2.8270	0.7365	0.0001
*HYPERSALIVATION*	${\hat{\beta }}_{5}$	1.7935	0.6005	0.0028
*PARALYZED*	${\hat{\beta }}_{6}$	2.0957	0.7374	0.0045
*DEAD*	${\hat{\beta }}_{7}$	1.2737	0.5950	0.0323
*ASS*_1_	${\hat{\beta }}_{8}$	5.8251	1.0549	<0.0001
*ASS*_2_	${\hat{\beta }}_{9}$	4.9193	1.1235	<0.0001

**Table 4:** Odd ratio estimates and 95% Confidence Limits (LCL, UCL) with Model II.

Variable	Odd Ratio	LCL	UCL
*BITES*	3.446	1.151	10.314
*AGE*_1_	7.329	1.798	29.871
*AGE*_2_	3.618	1.295	10.108
*AGE*_3_	16.895	3.989	71.554
*HYPERSALIVATION*	6.011	1.853	19.501
*PARALYZED*	8.131	1.916	34.504
*DEAD*	3.574	1.114	11.472
*ASSE*_1_	338.702	42.844	>999.999
*ASSE*_2_	136.910	15.141	>999.999

Model II:

$$log\left(\frac{p}{1-p}\right)={\hat{\beta }}_{0}+{\hat{\beta }}_{1}BITES+{\hat{\beta }}_{2}AG{E}_{1}+{\hat{\beta }}_{3}AG{E}_{2}+{\hat{\beta }}_{4}AG{E}_{3}+{\hat{\beta }}_{5}HYPERSALIVATION+{\hat{\beta }}_{6}PARALYZED+{\hat{\beta }}_{7}DEAD+{\hat{\beta }}_{8}ASS{E}_{1}+{\hat{\beta }}_{9}ASS{E}_{2}$$

### Sensitivity and specificity analysis, and model validation

Model I showed high sensitivity, but low specificity when a probability level of 0.01 was used as a cutoff value to classify the dog as having rabies (SE = 97.9%, SP = 66.3%) (Table 5). Sensitivity remained above 80% and specificity improved when probability cutoff value was below 0.06. These associations of sensitivity and specificity were similar when evaluated with the validation dataset.

**Table 5:** Sensitivity (SE, %) and specificity (SP, %) for predicting rabies in dogs in HARSP 2014–2016, using Models I and II.

Prob. Level	Model I				Model II			
	2014–2015 (Fit)		2016 (Validation)		2014–2015 (Fit)		2016 (Validation)	
	SE	SP	SE	SP	SE	SP	SE	SP
0.01	97.92	66.27	100.00	66.45	97.92	91.77	100.00	0.00
0.02	89.58	88.46	83.33	95.94	97.92	91.92	100.00	0.00
0.03	89.58	88.46	83.33	95.94	97.92	91.92	100.00	0.00
0.04	89.58	88.46	83.33	95.94	97.92	92.80	100.00	56.41
0.05	81.25	91.55	83.33	97.01	97.92	92.80	100.00	56.41
0.06	79.17	91.62	83.33	97.01	97.92	92.95	100.00	56.41
0.07	79.17	91.62	83.33	97.01	97.92	92.95	100.00	56.41
0.08	66.67	96.11	66.67	98.08	97.92	92.95	100.00	56.41
0.09	60.42	97.65	50.00	99.15	95.83	96.03	100.00	56.41
0.10	58.33	97.65	50.00	99.15	95.83	96.03	100.00	56.41
0.20	50.00	98.68	50.00	99.57	89.58	97.43	100.00	95.94
0.30	45.83	99.12	16.67	99.57	72.92	98.46	100.00	97.44
0.40	45.83	99.49	16.67	99.57	64.58	99.12	100.00	97.44
0.50	45.83	99.56	16.67	99.57	58.33	99.41	83.33	97.44
0.60	45.83	99.56	16.67	99.57	43.75	99.63	83.33	97.44
0.70	35.42	99.78	16.67	100.00	35.42	99.78	83.33	97.44
0.80	25.00	99.78	0.00	100.00	27.08	99.78	83.33	97.44
0.90	14.58	99.78	0.00	100.00	16.67	99.85	66.67	97.86
1.00	0.00	100.00	0.00	100.00	0.00	100.00	0.00	100.00

Model II showed high sensitivity and specificity when a probability level of 0.10 or lower was used to classify dogs as rabid (SE >95%, SP >91%). Model II performed better on the validation dataset, with a sensitivity of 100% up to the 0.4 probability level.

### Model application

Table 6 shows the rabies status for cases without a confirmed outcome (i.e. clinical case definition of probable rabies and suspect rabies cases) in the periods of 2014–2015 and 2016, predicted with Models I and II, respectively. When utilizing the assessor’s model (Model II) and using a probability level of 0.1 as a cutoff value to define rabies status, 81% of dogs with a case status of ‘probable’ and 45% of dogs with a case status of ‘suspect’ would have been defined as rabid.

**Table 6:** Rabies status of the probable rabies cases (PRCs) and suspect rabies cases (SRCs) determined by Models I and II.

Probability Level	Predicted Rabies Status	Model I				Model II			
		2014–2015		2016		2014–2015		2016	
		PRCs (43)	SRCs (265)	PRCs (10)	SRCs (105)	PRCs (43)	SRCs (265)	PRCs (10)	SRCs (105)
0.05	Non-Rabid	20 (47%)	179 (68%)	6 (60%)	76 (72%)	8 (19%)	145 (55%)	0 (0%)	0 (0%)
	Rabid	23 (53%)	86 (32%)	4 (40%)	29 (28%)	35 (81%)	120 (45%)	10 (100%)	105 (100%)
0.06	Non-Rabid	21 (49%)	179 (68%)	6 (60%)	76 (72%)	8 (19%)	145 (55%)	0 (0%)	0 (0%)
	Rabid	22 (51%)	86 (32%)	4 (40%)	29 (28%)	35 (81%)	120 (45%)	10 (100%)	105 (100%)
0.07	Non-Rabid	21 (49%)	179 (68%)	6 (60%)	76 (72%)	8 (19%)	145 (55%)	0 (0%)	0 (0%)
	Rabid	22 (51%)	86 (32%)	4 (40%)	29 (28%)	35 (81%)	120 (45%)	10 (100%)	105 (100%)
0.08	Non-Rabid	28 (65%)	259 (98%)	7 (70%)	101 (96%)	8 (19%)	146 (55%)	0 (0%)	0 (0%)
	Rabid	15 (35%)	6 (2%)	3 (30%)	4 (4%)	35 (81%)	119 (45%)	10 (100%)	105 (100%)
0.09	Non-Rabid	29 (67%)	259 (98%)	8 (80%)	101 (96%)	8 (19%)	146 (55%)	3 (30%)	55 (52%)
	Rabid	14 (33%)	6 (2%)	2 (20%)	4 (4%)	35 (81%)	119 (45%)	7 (70%)	50 (48%)
0.10	Non-Rabid	29 (67%)	259 (98%)	8 (80%)	101 (96%)	8 (19%)	146 (55%)	3 (30%)	55 (52%)
	Rabid	14 (33%)	6 (2%)	2 (20%)	4 (4%)	35 (81%)	119 (45%)	7 (70%)	50 (48%)
0.20	Non-Rabid	31 (72%)	260 (98%)	8 (80%)	101 (96%)	19 (44%)	179 (68%)	5 (50%)	55 (52%)
	Rabid	12 (28%)	5 (2%)	2 (20%)	4 (4%)	24 (56%)	86 (32%)	5 (50%)	50 (48%)
0.30	Non-Rabid	31 (72%)	263 (99%)	9 (90%)	103 (98%)	19 (44%)	180 (68%)	6 (60%)	76 (72%)
	Rabid	12 (28%)	2 (1%)	1 (10%)	2 (2%)	24 (56%)	85 (32%)	4 (40%)	29 (28%)
0.40	Non-Rabid	31 (72%)	263 (99%)	10 (100%)	104 (99%)	31 (72%)	263 (99%)	7 (70%)	78 (74%)
	Rabid	12 (28%)	2 (1%)	0 (0%)	1 (1%)	12 (28%)	2 (1%)	3 (30%)	27 (26%)
0.50	Non-Rabid	34 (79%)	263 (99%)	10 (100%)	104 (99%)	35 (81%)	263 (99%)	8 (80%)	103 (98%)
	Rabid	9 (21%)	2 (1%)	0 (0%)	1 (1%)	8 (19%)	2 (1%)	2 (29%)	2 (2%)

Considering the national rabies control program in Haiti, which typically assesses 5,000 people each year for dog bites, a 5% probability that the biting dog had rabies, and where all victims are treated as exposed with a 4-dose vaccination series ($12 per dose), total human rabies vaccine costs used in Haiti would be valued at $240,000 USD. Provision of PEP at this level would prevent 45 human rabies deaths each year, nationally.

Assuming Model I parameters to guide the provision of PEP, at a tolerance level of 1% to approve release of PEP to the bite victim, 5.2 rabies-exposed persons would be inappropriately advised to delay PEP, resulting in 0.9 human rabies deaths each year. PEP would be correctly delayed to 3,148 persons bitten by non-rabid dogs. Overall PEP provision would be reduced 63%, resulting in a PEP cost-savings of $151,345 (Fig. 1). If the tolerance level were raised to 10%, under this scenario, 104 rabies-exposed persons would be incorrectly advised to delay PEP, resulting in 19 human rabies deaths. PEP provisions could be reduced 95%, resulting in PEP cost-savings of $227,642. The cost-savings per human rabies death using Model I at tolerance levels of 1% and 10% are $161,694 and $12,358 USD, respectively (Fig. 3).

If Model II was used to inform PEP provision, incorporating the trained investigator’s assessment decision, the tolerance level could be increased to 8% resulting in similar human health outcomes as predicted in Model I (0.9 human rabies deaths) (Fig. 2). At a tolerance of 8%, PEP provision could be reduced 88%, resulting in a PEP cost-savings of $212,176. The PEP cost-savings per additional human rabies death under this scenario is $223,809 USD, or $44.76 in PEP cost-savings per bite investigation (Fig. 3).

## Discussion

Globally, tens of thousands of people die each year from rabies virus infection due to lack of appropriate post-exposure prophylaxis after a bite from a rabid dog. Increased access to PEP has been proposed as one means of reducing human rabies deaths, particularly when used in combination with mass dog vaccination strategies^20^. However, given that dog bites are a relatively common event in many countries, and the cause of dog bites is rarely because the animal is rabid, unfettered use of rabies PEP in the absence of a risk-based approach may lead to very high program costs and frequent supply shortages^10^. PEP delivery models that can reduce the unnecessary use of PEP while not increasing rabies risks for bite victims will likely result in more sustainable programs, which could potentially enable countries to shift resources from PEP-associated costs into programs for sustained dog vaccination programs. Several studies have explored the risk factors associated with rabid dogs, but all have so-far included the results of a quarantine period, which diminishes their value for the determination of risk at the time of initial rabies risk-assessment. This study shows that PEP use could be significantly reduced, while placing little-to-no added risk onto bite victims, in the context of an experienced IBCM program.

Rabies is an invariably fatal disease when PEP is not initiated in a timely manner. As such, many countries have developed policies in which PEP is initiated immediately when rabies is suspected, and then discontinued when the animal is proven rabies-free through quarantine or laboratory diagnostics. While this “start-stop” process ensures that few people (if any) with true exposures go untreated, it results in only minimal vaccine savings. Furthermore, many programs recommend that bite victims complete the vaccination series even after rabies is ruled out, so that the bite victim is unequivocally considered vaccinated if future exposures occur. Programs in which vaccine supplies are limited, or costs of PEP are hampering more effective control methods such as dog vaccination, must consider more efficient PEP-delivery systems. This study found that, under the situation of Haiti’s national rabies control program, accurate risk determinations could be made at the time of animal assessment, and that the risk to bite victims could be completely negated when PEP is reconsidered if the dog developed signs of illness during the observation period.

Two models were developed to predict the probability that a biting dog has rabies, one considering only standardized data routinely collected during rabies investigations and a second that additionally considered the trained bite investigator’s subjective opinion on whether or not the dog was rabid. The model that included the investigator’s opinion was more sensitive and specific, which allowed for selection of a higher tolerance level, increased PEP savings, and a very small risk that a bite victim was incorrectly informed to delay PEP. It is possible that the data routinely collected during rabies investigations in Haiti do not include factors that can more accurately predict the rabies status of biting dogs, and that rabies investigators are able to discern factors associated with the bite event that are not otherwise captured in the case investigation form. Most rabies investigators in Haiti undergo a minimum 1-week training and spend 1-month under close supervision by a program manager. Many of the investigators in Haiti’s IBCM program, which incorporates most data used to develop Model II, have been conducting rabies investigations since 2013. A program utilizing less experienced rabies investigators may have less success reproducing the accuracy observed with Haiti’s program. When highly qualified staff are available, Model II considering their assessment decision should be used to quantify the risk of rabies in biting dogs. If no trained professionals are available, we recommend using Model I.

Considering the IBCM risk-based approach to PEP provision, all models found a small risk that an incorrect determination could be made, and persons may be incorrectly advised to delay initiation of rabies PEP. This can be overcome by developing an algorithm in which PEP is reconsidered based on quarantine outcome or diagnostic results (Fig. 4). Medley *et al*.^14^ found that 100% of rabid dogs assessed through Haiti’s national rabies surveillance program died within 4 days of being placed into quarantine. Therefore, in the absence of a severe bite wound to the head (for which PEP should not be delayed), an algorithm which delays PEP while low-risk animals are under quarantine would identify incorrectly classified animals within approximately 4 days of the investigation. Under this algorithm, PEP would still be initiated within 10-days of the bite event, likely negating any risk of rabies in the exposed individuals. Future algorithms which are considered for rabies PEP guidance should undergo a cost benefit analysis similar to what was conducted in this study.

The World Health Organization recommends PEP be initiated immediately for exposures to a rabies suspect animal after a risk assessment has been conducted to establish the case status of the offending animal^10^. The case definition for “rabies suspect” includes clinical factors and circumstances of the bite event. Clearly, not all bites are from dogs satisfy this case definition for “rabies suspect”. This study found that rabid dogs in Haiti were significantly more likely to display clinical signs of hypersalivation and paralysis, and rabid dogs were much more likely to be found deceased at the time of investigation (often killed by community members before the investigator arrived). These clinical signs were detected by well-trained investigators. In the absence of such capacity, it may be difficult to determine if biting dogs fulfilled the case definition and as a result PEP could be over-prescribed, or incorrectly denied. This study found that utilizing a risk-based approach for PEP provision could save up to $44 in PEP-associated costs per rabies investigation conducted. Rabies programs that lack the technical capacity to conduct IBCM should consider both the positive health impacts for bite victims as described by Etheart *et al*.^9^, as well as these beneficial economic impacts when considering investment into IBCM program development.

This model may not be applicable to other rabies programs, as the technical capacity of investigators as well as the cultural sensitivities of the community may impact the course of an investigation. For example, in Haiti there is little cultural aversion to killing a dog that poses a threat to community members (i.e. a suspect rabid dog), and this is likely the reason that many rabid dogs are found dead at the time of investigation. However, some cultures (i.e. India, Thailand) are more averse to killing animals, even when they are sick or a danger to the community^21–23^. In these situations, it is unlikely that such strong associations between finding an animal deceased and the animal having rabies will be observed. Inadequately experienced investigators as well as differences in rabies virus reservoir species may also change the recognition and development of clinical signs in rabid animals.

This model was derived from a dataset of biting dogs with a known outcome: rabid or non-rabid. However, in Haiti up to 50% of dogs involved in a bite event do not have a known outcome. They are never located by investigators, are too decomposed for testing, are lost during quarantine, and numerous other circumstances that result in an inability to confirm the rabies status in the dog. This model was used to estimate the risk in dogs that are classified as “probable” or “suspect” based on a clinical case definition. Model II was used to validate this clinical case definition. It was found that the risk of rabies in animals identified as “probable” is very high (81%), and the risk in dogs identified as “suspect” is still a concerning 45%. This represents a very high risk of rabies in these dogs that are not available for assessment, and supports the proposed algorithm which calls for PEP provision when the biting animal is not available for assessment.

Rabies remains the world’s deadliest zoonotic disease, despite a century of available human and animal vaccines and decades of national rabies elimination case-examples. Global elimination of dog-mediated human rabies deaths is likely to require improved access and adherence to human rabies vaccines. Increasing access to human rabies vaccine is thought to be much more feasible on a global scale under the context of an IBCM-strategy [20]. This study has shown that IBCM can be successful enacted in a resource limited setting and has the potential to significantly reduce human rabies vaccine costs; costs that will ideally be re-allocated to dog vaccination activities to achieve permanent canine rabies elimination.
